# Right-Sided Congenital Diaphragmatic Eventration in a Neonate: Diagnostic Laterality and the Role of Advanced Imaging

**DOI:** 10.7759/cureus.115145

**Published:** 2026-08-25

**Authors:** Ayesha Zubair, Mohammad Shaiq Mahmood, Muhammad Mansoor Ijaz, Muhammad Waqas Basharat, Awais Nasir

**Affiliations:** 1 Pediatric Medicine, District Headquarters Hospital, Chiniot, PAK; 2 Pediatrics, Allied Hospital, Faisalabad, PAK; 3 Pediatrics and Child Health, Faisalabad Medical University, Faisalabad, PAK; 4 Gynecology and Obstetrics, Addenbrooke's Hospital, Cambridge, GBR

**Keywords:** congenital diaphragmatic hernia, diaphragmatic laterality, hrct chest, respiratory distress, right hemidiaphragm

## Abstract

Congenital diaphragmatic eventration (CDE) is a rare pathology characterized by elevation of the hemidiaphragm resulting from abnormal development of diaphragmatic musculature. Its clinical and radiographic presentation can closely mimic more common and more immediately dangerous neonatal conditions. Although the reported laterality of CDE varies among published series, we present a male neonate with respiratory distress initially suspected to have congenital pneumonia or sepsis who was subsequently diagnosed with right-sided CDE. Diagnosis was established after escalation of imaging techniques, requiring high-resolution computed tomography (HRCT) of the chest to distinguish CDE from other pathologies. The patient was initially managed medically; however, surgical intervention was required to correct the underlying cause. This case addresses the importance of advanced cross-sectional imaging techniques in accurately identifying CDE and its laterality, thereby distinguishing it from its more common mimics.

## Introduction

Diaphragmatic eventration is an uncommon developmental abnormality in which the diaphragm remains anatomically continuous but is abnormally elevated because normal muscle fibers are replaced, partially or completely, by thin fibroelastic tissue [1,2]. It accounts for roughly five percent of all diaphragmatic anomalies and may be congenital, arising from failure of muscularization of the pleuroperitoneal membrane during fetal development, or acquired, most often secondary to phrenic nerve injury from birth trauma, mediastinal pathology, or iatrogenic causes such as cardiothoracic surgery [1,3]. Reported incidence figures vary widely across series, ranging from roughly one in 1,400 to as high as one to three per 1,000 live births, reflecting both the rarity of the condition and the number of cases that go undetected because they are asymptomatic [3,4]. The right hemidiaphragm is affected somewhat more often than the left, and males appear to be affected more frequently than females [2].

Clinical presentation is highly variable and depends on the extent of diaphragmatic weakness and the degree of restriction it places on the underlying lung. Many cases are discovered incidentally, but neonates with significant eventration typically present with respiratory distress, tachypnea, and feeding difficulties, occasionally progressing to cyanosis and paradoxical chest wall movement on the affected side [1,4]. Because eventration can closely mimic congenital diaphragmatic hernia both clinically and on plain radiography, distinguishing between the two is critical, as their management differs substantially; cross-sectional imaging such as ultrasonography or CT is often required to confirm an intact diaphragm and rule out true herniation of abdominal viscera [2,4]. Associations have been described with pulmonary sequestration, congenital heart disease, chromosomal anomalies, and intestinal malrotation [3]. Management ranges from conservative respiratory support in mild or asymptomatic cases to surgical plication of the diaphragm in infants with significant respiratory compromise [1,4]. We report the case of a five-day-old male neonate who presented with respiratory distress and was ultimately diagnosed with congenital eventration of the right hemidiaphragm, successfully managed with surgical plication.

## Case presentation

A five-day-old male neonate, born to non-consanguineous parents and a resident of Okara, Pakistan, presented to the emergency room with a three-day history of respiratory distress and poor feeding. He was a term baby delivered via normal vaginal delivery. The antenatal period was unremarkable. There was no fever, foul-smelling or discolored liquor, or maternal pyrexia before or after birth. The perinatal period was uneventful, with no history of delayed cry, cyanosis, or moaning at birth. The baby remained well for the first two days of life before the onset of symptoms. On examination, the infant appeared sick, with visible signs of respiratory distress. Vital signs revealed a pulse rate of 154 beats per minute, a respiratory rate of 68 breaths per minute, a temperature of 98°F, and a systolic blood pressure of 60 mmHg. There was no pallor, cyanosis, or edema, and no gross congenital anatomical anomaly was noted. Anthropometric measurements were as follows: occipitofrontal circumference 34 cm, length 49 cm, and weight 2.6 kg.

Systemic examination revealed a normal-shaped chest with the apex beat palpable in the fourth intercostal space, one centimeter lateral to the midclavicular line; both heart sounds were audible with no added sounds. On respiratory examination, there was reduced air entry on the right side with an area of dullness on percussion, along with subcostal and intercostal recessions, though vesicular breathing was otherwise normal with no added sounds. The abdomen was soft, non-distended, and non-tender, with audible bowel sounds; no viscera were palpable. Neurological examination was unremarkable, with a flat and open anterior fontanelle, normal tone, and bilaterally equal and reactive pupils.

Given the presentation, the initial differential diagnosis included congenital pneumonia, sepsis, congenital heart disease, and congenital lung malformation. Baseline investigations were sent (Table 1).

**Table 1:** Baseline investigations Hb: hemoglobin; TLC: total leukocyte count; CRP: C-reactive protein

Parameter	Patient value	Reference range
Hb	18.2 g/dL	15-24 g/dL
TLC	10,300 /mm^3^	9100-34,000 /mm^3^
Platelets	214,000 /mm^3^	84,000-478,000 /mm^3^
CRP	Qualitative (negative)	Qualitative (positive/negative)
Sodium	140 mmol/L	133-146 mmol/L
Potassium	3.6 mmol/L	3.2-5.5 mmol/L
Chloride	108.1 mmol/L	97-110 mmol/L
Bicarbonate (Arterial)	23 mEq/L	21-28 mEq/L
pH	7.39	7.35-7.45
PCO_2_	42 mmHg	35-45 mmHg
PO_2_	62 mmHg	50-95 mmHg
Urea	32 mg/dl	3-12 mg/dL
Creatinine	0.9 mg/dl	0.8-1 mg/dL

**Figure 1:**
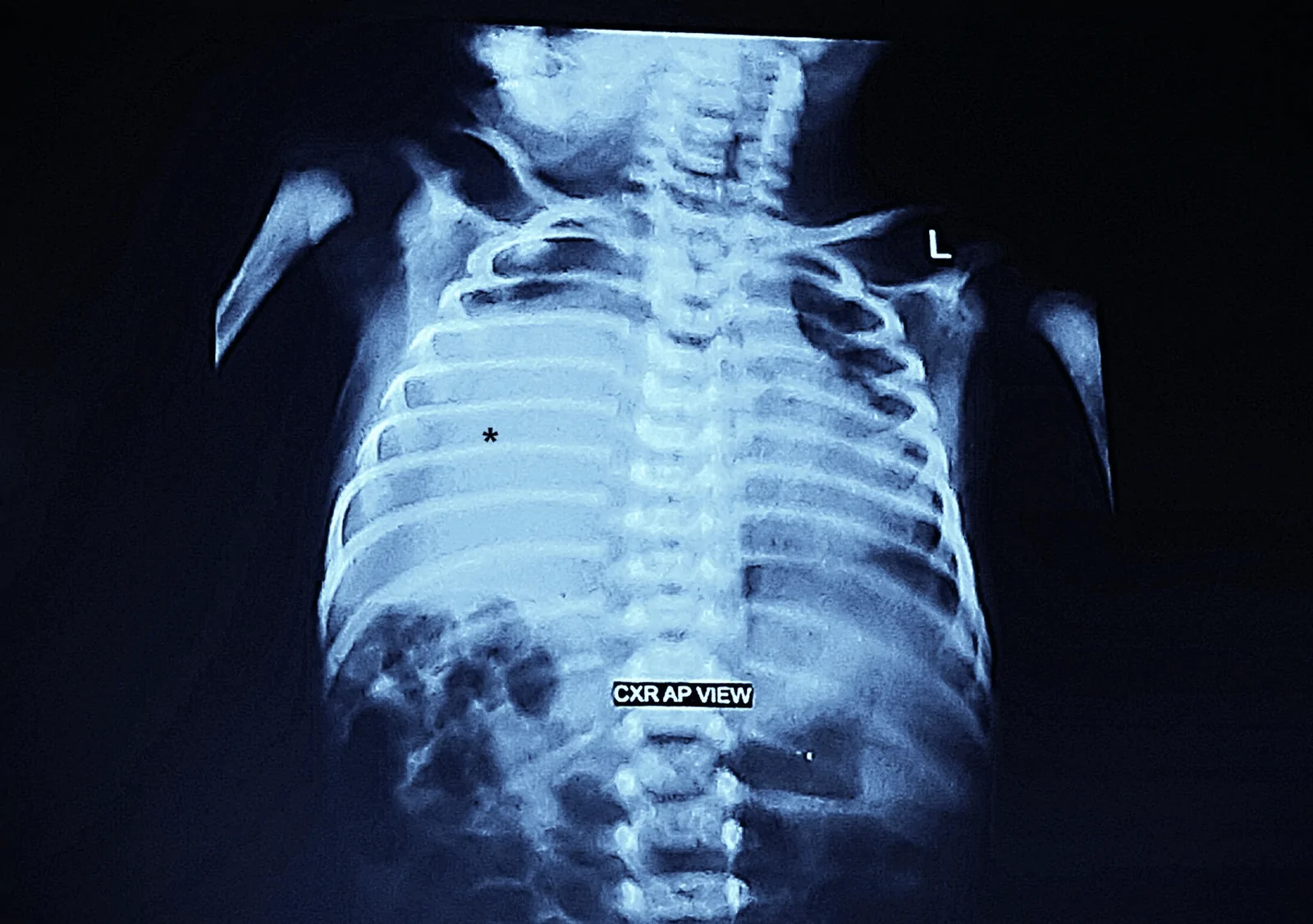
Chest X-ray (anteroposterior view) Marked elevation of the right hemidiaphragm with right lower lung compression (asterisk), consistent with right diaphragmatic eventration.

A chest radiograph (anteroposterior view) was obtained (Figure 1), which prompted a revision of the differential diagnosis to include diaphragmatic hernia, diaphragmatic eventration, congenital pulmonary airway malformation, and pulmonary sequestration.

**Figure 2:**
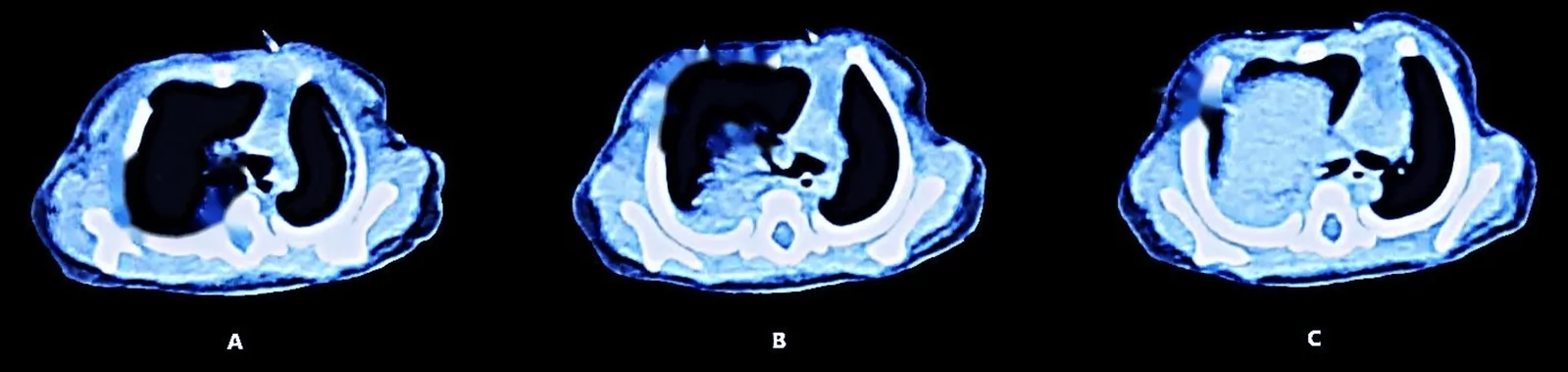
Imaging findings A: superior displacement of the liver and mild right lung compression; B: marked smooth elevation of the right hemidiaphragm with preservation of diaphragmatic continuity and cephalad displacement of the liver beneath the intact diaphragm; C: associated compressive atelectatic changes involve the right middle and lower lung lobes, with mild leftward mediastinal displacement. No diaphragmatic defect or herniation of abdominal viscera is identified

Echocardiography excluded significant structural cardiac disease, while abdominal ultrasonography assessed associated abdominal abnormalities. Because sonography did not adequately characterize the entire diaphragmatic contour or confidently exclude a diaphragmatic defect, CT of the chest was subsequently performed for definitive anatomical assessment and characterization of the diaphragmatic abnormality and led to the definitive diagnosis (Figure 2).

The neonate was managed with an intravenous line and supplemental oxygen inhalation and was kept nil per oral initially, supported with intravenous fluids and first-line antibiotics (ampicillin and cefotaxime). Following confirmation of the diagnosis, a referral was made to the pediatric surgery department on the eighth day of life. The symptoms did not improve with conservative management, and therefore the neonate underwent surgical plication of the right hemidiaphragm the following day. The postoperative course was uneventful, and the baby was monitored in postoperative care for three days before being discharged in stable condition.

## Discussion

Congenital diaphragmatic eventration (CDE) is a rare anomaly of the diaphragm, with Konstantinidi et al. reporting an incidence of approximately one in 10,000 live births in their pooled systematic review encompassing 204 cases [1]. This rarity, combined with a clinical presentation that overlaps substantially with far more common and immediately dangerous neonatal conditions like congenital pneumonia, sepsis, congenital heart disease, and congenital diaphragmatic hernia (CDH), makes CDE a diagnosis that is easily missed or delayed unless actively considered [1,2]. In our patient, the initial differential diagnosis reflected exactly this overlap, and the diagnosis of CDE was only established after a stepwise escalation of imaging from plain chest radiography to ultrasonography, echocardiography, and finally high-resolution computed tomography (HRCT).

An important and somewhat underappreciated aspect of CDE is its laterality. Classic teaching, dating to older case series, holds that the left hemidiaphragm is more frequently affected than the right, a pattern attributed to relative protection and reinforcement of the right hemidiaphragm by the underlying liver [3]. Our patient, however, presented with right-sided eventration, which, while less classically taught, is far from an isolated finding in the more recent literature. Notably, Konstantinidi et al.'s own pooled analysis of 204 cases found a predominance of right-sided eventration, contrary to the left-sided pattern traditionally described in older texts [1]. This finding has since been echoed in larger contemporary pediatric surgical cohorts: Wu et al., reporting 177 cases treated over 12 years at a single institution, and more recently Heng et al., in a 22-center French multicentric cohort of 139 children published in 2025, both found right-sided eventration to be more common than left-sided, occurring in roughly two-thirds of cases in the latter series [4,5]. This apparent shift between classic teaching and more recent, larger pooled data suggests that right-sided predominance may be more representative of the true distribution of CDE than has traditionally been assumed, and our patient's presentation is therefore consistent with this more recent body of evidence rather than an exception to it.

This diagnostic overlap between CDE and CDH is compounded by the fact that plain radiography alone is frequently insufficient to reliably distinguish the two. Several reported cases of CDE have been initially misdiagnosed as CDH on frontal chest radiographs, with the correct diagnosis only reached after additional imaging or surgical exploration [6,7]. A recent Cureus case report described exactly this scenario in an infant whose frontal radiograph suggested herniation, but whose lateral chest film and subsequent surgical findings confirmed intact, elevated right-sided CDE [7]. A retrospective comparison of 78 CDH and 20 CDE cases similarly found that computed tomography meaningfully outperformed plain radiography in confirming diaphragmatic integrity and excluding herniation [8]. This is consistent with our own diagnostic pathway, in which CT images demonstrated marked smooth elevation of the right hemidiaphragm with preservation of diaphragmatic continuity and cephalad displacement of the liver beneath the intact diaphragm. Associated compressive atelectatic changes involved the right middle and lower lobes, with mild leftward mediastinal displacement. No diaphragmatic defect or herniation of abdominal viscera was identified, thereby favoring eventration over CDH.

Antenatal imaging has increasingly been explored as a means of establishing the diagnosis before birth. Pleural or pericardial effusion on prenatal ultrasonography has been proposed as a marker favoring eventration over true herniation, and fetal magnetic resonance imaging has been used to directly visualize the diaphragmatic dome as a continuous, thin hypointense line in cases of eventration, a feature not seen when true herniation is present [9,10]. While our patient's antenatal course was unremarkable and the diagnosis was made postnatally, these tools illustrate a broader shift toward earlier, imaging-driven diagnosis of diaphragmatic anomalies. Unusual anatomical variants of CDE have also relied heavily on cross-sectional imaging for diagnosis, including a case of right-sided eventration with an intrathoracic ectopic kidney, and a case in which the heart was noted on the left while the diaphragm was elevated on the right, both illustrating how CT and related imaging can reveal atypical presentations of an already uncommon condition [11,12].

Management of CDE is guided primarily by the degree of respiratory compromise. Mild or asymptomatic eventration may be managed conservatively, whereas infants with significant respiratory distress, as in our patient, generally require surgical plication [2,13]. A single-institution series of 125 children found thoracoscopic plication to be associated with shorter operative time and faster recovery compared with open surgery, though open thoracotomy remains a well-established approach, particularly in smaller neonates or resource-limited settings [13]. A separate series restricted to infants under three months of age similarly found thoracoscopic plication associated with shorter hospital stays and fewer early complications compared with open thoracotomy, though open surgery remained effective and safe [14]. Bilateral CDE has also been successfully managed thoracoscopically in staged fashion even in the neonatal period, further supporting the feasibility of minimally invasive approaches where expertise is available [15,16]. In our patient, because clinically significant respiratory distress persisted despite initial supportive management and was attributable to marked diaphragmatic elevation with ipsilateral lung compression, surgical plication was undertaken on the ninth day of life. The postoperative course was uneventful [4].

Broader outcome data reinforce the importance of accurate diagnosis. A cohort comparing surgically treated CDE and CDH patients found that infants with a true diaphragmatic defect (CDH) had significantly lower one-year survival than those with eventration, with meaningful differences in complication profile and postoperative care intensity between the two groups [17]. Taken together, this case reinforces both the rarity of CDE and the evolving understanding of its laterality and underscores that advanced imaging, used alongside a high index of clinical suspicion, remains essential in correctly distinguishing CDE from its more common mimics.

## Conclusions

CDE is a rare cause of neonatal respiratory distress that can closely mimic more common and more immediately dangerous conditions, particularly CDH, on initial clinical and radiographic assessment. This case highlights that right-sided eventration, though traditionally considered less common than left-sided disease, is increasingly recognized in larger contemporary series and should be considered even when classic teaching suggests otherwise. A high index of clinical suspicion combined with escalation to advanced cross-sectional imaging, such as HRCT, was essential in reaching an accurate diagnosis in our patient and in guiding timely surgical management. Early recognition and appropriate surgical intervention, as demonstrated by this case, can result in excellent clinical outcomes even in neonates presenting with significant respiratory compromise.
